# Biological Implications of the Intrinsic Deformability of Human Acetylcholinesterase Induced by Diverse Compounds: A Computational Study

**DOI:** 10.3390/biology13121065

**Published:** 2024-12-19

**Authors:** Ysaías J. Alvarado, Lenin González-Paz, José L. Paz, Marcos A. Loroño-González, Julio Santiago Contreras, Carla Lossada, Alejandro Vivas, Yovani Marrero-Ponce, Felix Martinez-Rios, Patricia Rodriguez-Lugo, Yanpiero Balladores, Joan Vera-Villalobos

**Affiliations:** 1 Laboratorio de Química Biofísica Experimental y Teórica (LQBET), Instituto Venezolano de Investigaciones Científicas (IVIC), Centro de Biomedicina Molecular (CBM), Maracaibo 4001, Zulia, República Bolivariana de Venezuela; alvaradoysaias@gmail.com (Y.J.A.); paty851@gmail.com (P.R.-L.); 2 Laboratorio de Modelado, Dinamica y Bioquímica Subcelular (LMDBS), Instituto Venezolano de Investigaciones Científicas (IVIC), Centro de Biomedicina Molecular (CBM), Maracaibo 4001, Zulia, República Bolivariana de Venezuela; lossadacarla@gmail.com (C.L.); alejandro.j.vivas@gmail.com (A.V.); 3 Departamento Académico de Química Inorgánica, Facultad de Química e Ingeniería Química, Universidad Nacional Mayor de San Marcos, Lima 15081, Peru; 4 Departamento Académico de Fisicoquímica, Facultad de Química e Ingeniería Química, Universidad Nacional Mayor de San Marcos, Lima 15081, Peru; mloronog@unmsm.edu.pe; 5 Departamento Académico de Química Orgánica, Facultad de Química e Ingeniería Química, Universidad Nacional Mayor de San Marcos, Lima 15081, Peru; jsantiagoc@unmsm.edu.pe; 6 Facultad de Ingeniería, Universidad Panamericana, Augusto Rodin 498, Insurgentes Mixcoac, Benito Juárez, Ciudad de México 03920, México or ymarrero@usfq.edu.ec (Y.M.-P.); fmartin@up.edu.mx (F.M.-R.); 7 Grupo de Medicina Molecular y Traslacional (MeM&T), Colegio de Ciencias de la Salud (COCSA), Universidad San Francisco de Quito (USFQ), Escuela de Medicina, Edificio de Especialidades Médicas, Diego de Robles y vía interoceánica, Quito 170157, Ecuador; 8 Laboratorio de Física de la Materia Condensada, Instituto Venezolano de Investigaciones Científicas (IVIC), Apartado 20632, Caracas, República Bolivariana de Venezuela; yanpiero@gmail.com; 9 Laboratorio de Análisis Químico Instrumental (LAQUINS), Facultad de Ciencias Naturales y Matemáticas, Departamento de Química y Ciencias Ambientales, Escuela Superior Politécnica del Litoral, Guayaquil ECO90211, Ecuador; joarvera@espol.edu.ec

**Keywords:** Alzheimer’s disease, structural flexibility, AChE inhibitors

## Abstract

The enzyme acetylcholinesterase (AChE) plays a crucial role in the termination of nerve impulses by hydrolyzing the neurotransmitter acetylcholine (ACh). The inhibition of AChE has emerged as a promising therapeutic approach for the management of neurological disorders such as Lewy body dementia and Alzheimer’s disease. The potential of various compounds as AChE inhibitors was investigated. In this study, we evaluated the impact of natural compounds of interest on the intrinsic deformability of human AChE using computational biophysical analysis. Our approach incorporates classical dynamics, elastic networks (ENM and NMA), statistical potentials (CUPSAT and SWOTein), energy frustration (Frustratometer), and volumetric cavity analyses (MOLE and PockDrug). The results revealed that cyanidin induced significant changes in the flexibility and rigidity of AChE, especially in the distribution and volume of internal cavities, compared to model inhibitors such as TZ2PA6, and through a distinct biophysical-molecular mechanism from the other inhibitors considered. These findings suggest that cyanidin could offer potential mechanistic pathways for future research and applications in the development of new treatments for neurodegenerative diseases.

## Simple Summary

Acetylcholinesterase (AChE) is a key enzyme responsible for terminating nerve impulses by hydrolyzing the neurotransmitter acetylcholine (ACh). The inhibition of AChE has gained attention as a therapeutic strategy for neurological disorders including Lewy body dementia and Alzheimer’s disease. This study investigated the effects of natural compounds on the intrinsic deformability of human AChE through computational biophysical analysis, utilizing methods such as classical dynamics, elastic networks, statistical potentials, energy frustration, and volumetric cavity analyses. The findings indicate that cyanidin significantly alters the flexibility and rigidity of AChE, particularly affecting the distribution and volume of its internal cavities, in contrast to model inhibitors like TZ2PA6. This distinct biophysical-molecular mechanism demonstrated by cyanidin highlights its potential as a target for future research and the development of new treatments for neurodegenerative diseases.

## 1. Introduction

The enzyme known as acetylcholinesterase (AChE) plays a crucial role in the proper functioning of neurons through cholinergic pathways. AChE is primarily found in the synaptic spaces of both the central and peripheral nervous systems as well as in the membranes of red blood cells. Its main function is to halt nerve impulses by catalyzing the hydrolysis of acetylcholine (ACh), a neurotransmitter involved in various cognitive processes within the cholinergic system [1,2]. The inhibition of the AChE enzyme using specific inhibitors has emerged as a promising therapeutic approach for managing a range of neurological disorders including Lewy body dementia and Alzheimer’s disease (AD) [1,2]. However, the accumulation of ACh in synapses can lead to pathological conditions. This occurs when ACh receptors, namely nicotinic and muscarinic receptors, are excessively stimulated, resulting in neurological ailments such as depression, dizziness, headaches, nausea, breathing difficulties, and even sudden death [3].

AChE inhibitors (AChEIs) are employed in the treatment of these disorders to enhance cholinergic activity by increasing the levels of ACh in cholinergic synapses [3]. Nonetheless, apprehensions regarding the toxicity and adverse effects associated with synthetic substances have spurred the quest for novel, safe, and effective natural compounds. In this regard, plant phenolic compounds have gained significant attention as extensively studied natural products due to their diverse biological applications including the inhibition of AChE [4].

Indeed, numerous studies have indicated that huperzine A, a derivative of the Chinese herb *Huperzia serrata*, exhibits selectivity, potency, and reversibility as an inhibitor of AChE. Both preclinical research and clinical trials have demonstrated the potential therapeutic effect of huperzine A in AD treatment, although there is currently insufficient evidence to recommend its clinical use. Presently, huperzine A is utilized as a dietary supplement in certain countries. Moreover, our research group has recently investigated other natural compounds, such as cyanidin and resveratrol, for their diverse therapeutic properties, particularly their potential as anti-Alzheimer’s alternatives, given their ability to interact with and inhibit AChE [5,6,7].

Cyanidin, known scientifically as 3,5,7,3′,4′-pentahydroxyflavilium, 2-(3,4-dihydroxyphenyl)-3,5,7-trihydroxy-1-benzopyrylium, is a naturally occurring organic compound that falls under the categories of both flavonoids and anthocyanins. It serves as a pigment and is commonly found in a variety of fruits including blackberries, raspberries, grapes, cherries, blueberries, and even purple corn. Additionally, cyanidin can be found in fruits like apples and plums. The highest concentrations of this compound are typically found in the fruit peel [8]. Resveratrol, chemically known as 3,5,4′-trihydroxystilbene, belongs to the class of stilbenoids, which are natural phenolic compounds. It is classified as a phytoalexin, naturally synthesized by different plants, particularly in the skin of grapes, blueberries, raspberries, and blackberries [9].

The use of natural compounds such as resveratrol and cyanidin as potential AChE inhibitors is highly relevant in the development of therapies for neurodegenerative diseases like Alzheimer’s for several reasons. First, AChE inhibitors represent a key therapeutic strategy for enhancing cholinergic transmission in the brain, which may alleviate cognitive symptoms associated with Alzheimer’s [10,11]. Natural compounds offer a promising alternative to synthetic inhibitors due to their lower toxicity and greater availability [12,13]. Resveratrol and other polyphenols have demonstrated potential in inhibiting AChE, which could not only enhance cholinergic function, but also reduce beta-amyloid aggregation, a central pathological process in Alzheimer’s [10,12]. Furthermore, natural compounds often exhibit multiple mechanisms of action, which could provide additional benefits in modulating other pathological pathways involved in Alzheimer’s [14,15]. Current research even suggests that natural compounds may serve as chemical scaffolds for the development of new drugs with improved properties [11,13]. However, it is essential to note that while these compounds show promise in preclinical studies, further research is needed to establish a mechanistic foundation including studies of molecular interactions and conformational changes induced by ligands to support the progression to in vitro and clinical trials [15].

On the other hand, computational techniques complement experimental findings in the study of inhibitory compounds that may alter the conformation of their target proteins by providing a detailed understanding of the molecular interactions and conformational changes induced by ligands. These techniques enable the modeling and prediction of how inhibitors bind to their target proteins and how they affect the conformation of these proteins, which is crucial for the design of effective drugs. For instance, the use of Molecular Dynamics (MD) simulations and Markov state modeling has proven effective in capturing the inhibition efficiency of compounds [16]. Molecular docking simulations and free energy binding analysis are employed to identify potential binding sites and predict binding affinities consistent with experimental data [17]. Furthermore, the inherent flexibility or deformability of protein systems has been studied as a result of bound ligands. Understanding this intrinsic deformability, which is determined by their three-dimensional structures or the inter-residue contact topology, is crucial for comprehending the flexibility and dynamics of cooperative movements that are closely associated with the structural alterations observed during their biological functions [18,19,20,21,22].

This is why studying the intrinsic flexibility of clinically relevant proteins is a crucial factor in drug design, particularly when addressing the complexities of protein–ligand interactions. Understanding protein dynamics and their ability to adopt multiple conformations is essential for improving the accuracy in drug design. Traditionally, the “lock and key” model has been insufficient to explain the complexity of these interactions, leading to the adoption of more dynamic models such as “induced fit” [23,24]. The flexibility of proteins allows them to explore different conformational states, facilitating their adaptation to various ligands and environmental conditions. This is particularly relevant in drug discovery, as a protein’s ability to change conformation can reveal cryptic binding sites and allosteric regulation mechanisms that would not be evident in static structures [25] Furthermore, flexibility can influence the kinetics and thermodynamics of drug binding, affecting both affinity and cellular efficacy [26]. Computational techniques, such as MD simulations, have advanced to incorporate this flexibility into structure-based design, enhancing the estimation of binding affinities and facilitating the discovery of new lead compounds [27,28]. These techniques enable the modeling of conformational changes that occur before and during ligand binding, providing a more comprehensive view of the biomolecular interactions [29].

Moreover, intrinsic conformational movements play a crucial role in protein function and are generally energetically advantageous. These movements are conserved throughout evolution among orthologs. As a result, it has been observed that the sequences and structures of proteins, shaped by evolutionary processes, rely on their intrinsic dynamics to carry out their specific functions. These intrinsic dynamics can be influenced by various physicochemical perturbations including ligand-induced changes. Such changes have the potential to alter the preferred modes or conformations that the protein adopts during its functional processes. Particularly, the binding of ligands can regulate the intrinsic dynamics by modifying the energy landscape of the protein, often by influencing its curvature [22].

The intrinsic deformability of AChE has been extensively studied, revealing that the magnitude of its structural fluctuations can significantly increase when specific inhibitors bind to the primary entry channel leading to the active site. This binding effectively abolishes the enzyme’s activity toward natural substrates like acetylcholine. Exploring the intrinsic flexibility of AChE has also been studied by other authors in relation to synthetic compounds obtained through “click” chemistry, resulting in the development of exceptionally potent binding compounds [30,31,32]. This approach enables the design of inhibitors that elicit a pronounced “induced fit” response in AChE, leading to the exposure of previously solvent-hidden regions. Consequently, ligand-induced conformational rearrangements can facilitate the formation of enzyme complexes optimized for the inhibition of AChE. This can be achieved by modulating the transient movements associated with both the active and peripheral sites. These interactions can even occur in time-dependent conformations [30,31].

Efforts to investigate the intrinsic deformability of AChE have primarily utilized classical dynamics tools [30,31], and elastic network models (ENMs) [32,33]. These approaches have been employed to analyze the inherent conformational movements of this protein system in the presence and absence of ligands [19,34,35,36]. This type of comparative analysis is particularly valuable for two main reasons. Firstly, the conformational flexibility of target proteins poses a significant challenge in accurately modeling and describing protein–inhibitor interactions [37,38,39]. Secondly, in theoretical inhibition assays, it is essential to determine whether the observed conformational changes, beyond those naturally controlled by the protein for its functional interactions, could be induced by the inhibitor [40].

In order to tackle the challenges and considerations above-mentioned, it is important to highlight that a crucial element in the biophysical exploration of intrinsic conformational movements lies in the utilization of complementary analyses based on statistical energy functions or potentials. These functions describe local interactions along the protein chain, hydrophobic forces, and tertiary interactions. This approach has demonstrated its effectiveness in predicting and interpreting critical regions within proteins with respect to conformational changes, protein folding, and perturbations at complex interfaces [38,41,42].

Furthermore, the utilization of algorithms inspired by energy landscape theory provides a means to quantify the localization and distribution of energy within protein structures. This concept has been proven to be valuable in gaining a deeper comprehension of the biological behavior of proteins and the impact of perturbations on their intrinsic energy. This intrinsic energy, which is conserved, is closely linked to conformational changes and plays a significant role in understanding protein dynamics [43,44,45].

In this study, our objective was to conduct a comprehensive biophysical-computational comparative analysis to investigate the potential impact of natural compounds of interest on the intrinsic deformability of human AChE. To achieve this, we employed models based on classical dynamics, elastic networks, statistical potentials, energy frustration, and volumetric analysis. With this integrative approach, we aimed to assess the extent of conformational changes induced by several ligands on AChE. This protein system holds significant biomedical relevance, particularly in the context of neurodegenerative diseases.

## 2. Materials and Methods

### 2.1. Selected Molecules and Preparation from Databases for Docking, Theoretical Inhibition, and Molecular Dynamics (MD)

The crystal structure of human acetylcholinesterase (AChE) (EC3.1.17) (PDB: 4EY5) was obtained in PDB format from the RCSB Protein Data Bank (https://www.rcsb.org/, accessed on 1 September 2024) [46]. For this study, we considered protein structure chain A and two drugs: the polyphenol resveratrol (CID: 445154) and the flavonoid cyanidin (CID: 128861). The selection of these natural compounds was based on the many potential health benefits they have been reported to offer such as antioxidant, cardioprotective, neurological, anti-inflammatory, and anticancer activities, among others. In fact, recent published literature has demonstrated that they can defend against neurodegenerative diseases including the treatment of Alzheimer’s due to their ability to interact with and directly inhibit AChE [5,6,7]. Additionally, we included as controls various compounds that have shown in vitro experimental activity against AChE such as the alkaloid bis-(-)-8-demethylmaritidine (CID: 169450457) [47], and huperzine A (CID: 854026) [46] as well as the compound modeled using the click chemistry method TZ2PA6 (CID: 5289508) [32]. The inclusion of compounds like TZ2PA6 was based on the fact that it represents a model of novel compounds based on the time-dependent conformational remodeling of crystal complexes, and that they have shown strong binding, forming stable complexes governed by a highly reproducible thermodynamic equilibrium [32]. All compounds were obtained from PubChem (https://pubchem.ncbi.nlm.nih.gov/, accessed on 1 September 2024) in SDF format and converted to PDB format using OpenBabel-3.0 [48]. For visualization, the Molegro Molecular Viewer (MMV) [49] package was utilized, an open-access tool developed by Molexus that is advantageous for drug discovery. This tool allows for 3D visualization, interaction analysis, and is compatible with multiple formats. Likewise, the BIOVIA Discovery Studio Visualizer [50] was utilized, which is also an open-access package that offers advanced 3D visualization, molecular interaction analysis, simulations, and compatibility with multiple formats, among other features.

To explore the interaction between AChE and the compounds, we constructed complexes using directed molecular docking based on the coordinates of the previously reported binding pocket [47]. To carry out this task, we employed the DockThor tool (https://www.dockthor.lncc.br/, accessed on 1 September 2024), a machine learning-based molecular docking algorithm [51]. In order to enhance the accuracy, the program was executed with approximately 1 × 10^6^ evaluations per run, while default parameters were utilized for the remaining configuration settings. One of the advantages of using DockThor is that it provides additional steps for the automatic preparation of ligands including the addition of partial charges, hydrogen atoms, and the adjustment of rotatable bonds, among other parameters. During the preparation stage, multiple PDB files were generated from the most favorable poses predicted by DockThor, representing AChE with and without ligands bound. During the sampling and ligand pose generation process, the selection criterion was limited to the three most thermodynamically favorable binding poses (with the highest negative magnitudes). This approach allowed us to focus on the most feasible and thermodynamically favorable positions within the complexes.

Based on this criterion, the selected complexes underwent further analysis including calculations of inhibitory potency and MD. The inhibition constant for the binding of ligand to proteins ($K_{i}$) (in units of M) was obtained as:

(1) $$K_{i}=e^{\left(\frac{{-\Delta G}_{dock}}{RT}\right)}$$

The equation provided defines the binding affinity ($\Delta G_{dock}$) in kcal.mol^−1^, where *R* represents the universal gas constant, and *T* represents the absolute temperature. According to this equation, a higher $K_{i}$ value indicates weaker binding between the inhibitor and the protein, suggesting a higher likelihood of dissociation of the protein–inhibitor complex. To determine the IC_50_ values (concentration of the inhibitor required to achieve 50% inhibition), we utilized the IC50-to-Ki web tool (https://bioinfo-abcc.ncifcrf.gov/IC50_Ki_Converter/index.php, accessed on 5 September 2024) assuming competitive inhibition as recommended [39]. In this study, we assumed a hypothetical quantitative or stoichiometric substrate–inhibitor ratio of 1:1 in order to simulate the same amount of protein and inhibitors and thus avoid concentration-driven interactions, based on the assumption of structural similarities between the ligand and the substrate as previously suggested [39,52]. In contrast, the experimental inhibitory concentration values were obtained in molar expression, as described later for each of the compounds considered. MD simulations were conducted on the docking hits with two primary objectives: (1) to assess the relative stability of the ligand within the binding pocket, and (2) to explore the conformations with minimum energy and analyze their impact on the thermodynamic and structural stability of the complexes.

For each protein–ligand complex, a series of procedures were carried out to relax the MD system. This process consisted of three phases: relaxation, equilibrium, and sampling, following the recommended protocols [39]. The MD simulation was conducted in an explicit water system, where the system was represented by a cubic water box measuring 70 Å along each axis (X, Y, Z) and maintaining a minimum distance of 8 Å from the surface of the protein and protein–ligand complexes to the edge of the box at the beginning of the simulation. The aqueous system comprised 8016 water molecules, 32 Na^+^ ions, and 22 Cl^−^ ions, resulting in a molar density of Na/Cl = 2.76 × 10^−3^. Each MD system contained either a free protein species or the system bound to each respective docking ligand. The Amber99SB-ILDN force field was specifically utilized in this simulation, which is a refined version of the Amber99SB protein force field designed to enhance the accuracy of side chain torsional potentials by better matching experimental NMR data, particularly for amino acids with complex side chains, making it more suitable for simulating protein structures in MD simulations. Additionally, the TIP3P water model was employed, as it is widely recommended for the study of AChE [53,54,55,56]. Furthermore, the ligands were automatically described using GAFF from the myPresto package, which is a general Amber force field for organic molecules. GAFF is designed to be compatible with existing Amber force fields for proteins. Finally, both the topology files for the ligands and for the proteins were automatically generated using the myPresto package [57]. The system was neutralized, and water molecules were treated as rigid bodies, with periodic boundary conditions applied. The simulation was initiated using the steepest descent algorithm and the conjugate gradient energy minimization method, with position restrictions on the atoms of the protein–ligand complex. An initial 100 ps simulation was conducted, allowing water molecules to diffuse around the protein and reach equilibrium with the protein–ligand system. The PME method was used to calculate the electrostatic contribution to non-bonded interactions, with a cutoff of 14 Å. The NVT ensemble was applied for 100 ps to balance all systems and thermalize them at 300 K, followed by a second run of 100 ps using the NPT ensemble to equilibrate the system at 1 atm and 300 K. The SHAKE algorithm was applied to satisfy the link geometry constraints. The production step was performed using the output from the NPT ensemble as the initial configuration for an MD production series at a constant temperature of 300 K, with a total simulation time of 200 ns. Minimum energy structures were obtained every 20 ns as target structures for subsequent analyses. The system was neutralized as suggested to study the ligand–protein systems [39]. In addition, several MD simulation analyses were included, such as the RMSD (root mean square deviation), RMSF (root mean square fluctuation), Rg (radius of gyration), and interaction depletion, alongside comparisons to positive controls as recommended [39,58,59]. All MD simulations and additional adjustments were carried out using the myPresto package, which can be accessed at https://www.mypresto5.jp/en/, accessed on 1 September 2024.

Although DockThor is a robust tool, the formed complexes were further validated after the MD cycle through the analysis of binding free energy ($\Delta G_{bind}$). We employed the MM/PBSA (molecular mechanics Poisson–Boltzmann surface area) method, which utilizes a thermodynamic integration approach (Table S1). This validation strategy is based on the fact that, in terms of predicting protein–ligand binding modes, MM/PBSA re-scoring procedures with integrated truncated structures exhibited a success rate of >80% in the benchmark compared to other popular methods such as AutoDock Vina (~70%) [60]. Moreover, MM/PBSA is capable of outperforming other robust scoring functions like Glide SP (success rate of 58.6%), achieving overall success rates of approximately 74% [61]. The final snapshots of the minimized system were subjected to MM/PBSA rescoring, implemented in AMBER22. In line with the aforementioned suggestion, we selected 2500 frames from the last 20 ns of the MD simulation to compute the solvation free energy and molecular mechanics potential energy, which were used to estimate the overall binding affinity of the complexes of interest. The equations to calculate the MM/PBSA binding free energy as well as the detailed parameters for the MM/PBSA calculation procedures [60] can be found in the Supplementary Materials. The ΔG_MM/PBSA_ of the complexes was determined based on the frames extracted after the MD simulation. For visualization and analysis, we employed MMV and BIOVIA. For more details on the method, we recommend referring to [39].

### 2.2. Determination of Conformational Changes of Dynamized Complexes Using Statistical Potentials, Elastic Network Models, and Energy Frustration

#### 2.2.1. Statistical Potentials of Complexes

To determine conformational changes in dynamized complexes, we utilized the CUPSAT (http://cupsat.tu-bs.de/index.jsp, accessed on 15 September 2024) [62] and SWOTein (http://babylone.ulb.ac.be/SWOTein/, accessed on 15 September 2024) packages [63]. These algorithms are inspired by potential statistics and are specifically designed to study protein stability in relation to fluctuations in the energy landscape. CUPSAT (Cologne University Protein Stability Analysis Tool) is a powerful tool used for analyzing and predicting protein stability changes. It employs structural environment-specific atom potentials and torsion angle potentials to predict the unfolding free energy (ΔG) in kcal.mol^−1^ of the complexes. The prediction model of CUPSAT is built upon a dataset of over 4000 non-redundant protein structures with 50% sequence identity. The atomic potentials are derived from a radial pair distribution function and an atomic classification system. Boltzmann energy values are then calculated based on the distribution of radial pairs of amino acid atoms [62]. The details of the equations and theoretical foundations for calculating the energy functions of the CUPSAT model, which were derived from the mean force potentials and based on the inverse Boltzmann principle (establishing a close relationship between probability densities and energies), are shown in the Supplementary Materials.

SWOTein (Strengths and Weaknesses of prOTEINs) is a research tool that investigates how individual residues contribute to the overall folding free energy of proteins. It accomplishes this by utilizing statistical potentials derived from databases that incorporate structural elements including inter-residue distances. Positive contributions to free energy are interpreted as stability weaknesses, while negative contributions represent stability strengths. Statistical potentials have proven useful in studying various biophysical properties of proteins such as protein–protein interactions. The SWOTein model employs knowledge-based mean force potentials derived from datasets of experimentally solved 3D protein structures, employing the principles of the inverse Boltzmann law. The central objective of this model is to establish a relationship between the free energy of a given state and the probability of observing the system in that state. These statistical potentials are based on a coarse-grained representation of protein structures, focusing on the main-chain heavy atoms and the amino acid-dependent geometric average center of the side chains [41,63]. The details of the equations and theoretical foundations for calculating the energy functions of SWOTein model are shown in the Supplementary Materials.

By combining atomistic MD trajectories and minimum energy structures, we gain a comprehensive understanding of the dynamics and atomic fluctuations within a complex [64]. The low-frequency modes of the elastic network model (ENM) align closely with the dynamic modes or minimum energy structures predicted by MD simulations, exhibiting consistent directions, relative amplitudes of motion, and distances. As a result, several ENM-based approaches enable various applications including direct MD simulations and the incorporation of receptor flexibility in docking approaches [65].

#### 2.2.2. Analysis of Intrinsic Deformability Associated with AChE Conformational Changes

In this study, tools based on ENM, a technique known as coarse-grained (CG) normal mode analysis (NMA) [66,67], were considered. These tools offer a valuable approach for studying the vibrational dynamics of protein systems near their energy minimum state. In the ENM model, the protein structure is represented using a reduced set of atoms, specifically the Cα atoms, which are treated as nodes. The interactions between pairs of nodes are described by a single-term Hooke harmonic potential. ENM approaches utilize two methods for computing the cross-correlation of atomic motion. The first method, known as the linear cutoff ENM, assumes a force constant for pairwise interactions between Cα atoms within a specified cutoff distance. The second method, referred to as Kovacs-ENM, adjusts the force constant based on the distance between the interacting particles [64,68,69,70,71,72]. The details of the equations and theoretical foundations of ENM are shown in the Supplementary Materials.

Specifically, in this study, we utilized the SPECTRUS (SPECTral-based Rigid Units Subdivision) server (http://spectrus.sissa.it/#home, accessed on 20 September 2024) [73], which performs a decomposition of proteins or protein complexes into quasi-rigid domains. This decomposition is based on analyzing the fluctuations in distances between pairs of amino acids. To compare the functional dynamics of protein complexes with varying degrees of structural similarity, we employed the MD trajectories obtained under the aforementioned conditions. SPECTRUS utilizes an ENM approach, which considers the specific properties of each protein complex and its free energy landscape. This allows for the reliable reproduction of structural fluctuations [73]. Quasi-rigid domain decomposition methods are based on the concept that, for truly rigid bodies, the distances between any two constituent points remain constant during motion in space [73]. The details of the equations and theoretical foundations of NMA and SPECTRUS are shown in the Supplementary Materials.

In order to further explore and improve the comprehensiveness of our search for potential conformational changes in the complexes, we utilized the DynOmics-ENM server (https://dyn.life.nthu.edu.tw/oENM/, accessed on 20 September 2024). This server offers a dynamic analysis of biomolecular systems using two popular ENMs: the Gaussian network model (GNM) and anisotropic network model (ANM). By integrating these models, the server calculates the dynamics of structural coordinates. The DynOmics-ENM method considers various unique characteristics such as the environment, functional site prediction, and the reconstruction of coarse-grained deformed structures. For more detailed information about the method, we recommend referring to [74]. GNM and its extension ANM are ENMs that operate at a coarse-grained residue level. GNM focuses on predicting the relative magnitudes of fluctuations, while ANM goes a step further by predicting both the magnitudes and directionalities of collective motions. GNM results are generally more robust and are therefore preferred for evaluating square displacements in low-frequency modes. GNM is specifically used to calculate mean-square fluctuations and correlations between residue fluctuations. On the other hand, ANM is utilized to generate conformations that describe residue fluctuations based on the average X-ray structure, specifically in the principal directions of motion [72].

#### 2.2.3. Local Energy Frustration of Complexes

Proteins undergo biologically optimized folding processes to achieve stability and carry out their functions effectively. Consequently, it is expected to observe a certain level of energetic conflict within their local structures. These conflicts enable proteins to explore various conformations within their native set, ultimately contributing to the emergence of “biological function”. To gain insights into the configurational patterns, dynamics, and transitions of complex systems, an examination of frustration and its role in the functional dynamics of these complexes was conducted [45]. To quantify the degree of local protein frustration, we utilized the Frustratometer software v2 (http://frustratometer.qb.fcen.uba.ar/, accessed on 25 September 2024) [43,45]. This software is built on the energy landscape theory and provides a model for assessing protein frustration. The Frustratometer employs the associative memory, water mediated, structure and energy model (AWSEM), a non-additive and coarse-grained force field that can accurately predict protein and complex structures. AWSEM incorporates various factors including hydrogen bonding and a bioinformatics-based local structure bias term that considers the effects of neighboring bodies influenced by the local sequence. The local frustration index provided by the Frustratometer measures the contribution of a residue or pair of residues to the overall energy of each structure, particularly when they deviate from their native positions. This index effectively captures the energy variations associated with the molten globule conformations of the polypeptide chain. The local frustration index is particularly valuable for studying tertiary structures and allows for the analysis of energy variations by manipulating parameters such as the distances and densities of the interacting amino acids. For more detailed information about the methodology, we recommend referring to the references provided [43,45]. The details of the equations and theoretical foundations of the Frustratometer are shown in the Supplementary Materials.

#### 2.2.4. Volumetric Analysis of Internal Cavities

To determine the changes in the internal cavities of the AChE protein in each complex, the MOLE 2.5 tool (https://mole.upol.cz/, accessed on 30 September 2024) was used. This tool specializes in the rapid and fully automated localization and characterization of internal cavities in (bio)macromolecular structures [75]. Additionally, the PockDrug tool (https://pockdrug.rpbs.univ-paris-diderot.fr/cgi-bin/index.py?page=Home, accessed on 30 September 2024) was employed to predict pocket druggability or the probability of a drug-like molecule interacting with a specific cavity. This tool provides consistent pharmacological results using different pocket estimation methods. It is robust with respect to pocket boundaries and estimation uncertainties, making it efficient even for pockets that are difficult to estimate. It clearly distinguishes druggable pockets from non-druggable ones using different estimation methods [76]. Furthermore, the web tool CavityPlus (http://www.pkumdl.cn:8000/cavityplus/index.php#/, accessed on 30 September 2024) was also used for the visualization of the internal cavities. It features a CAVITY module that generates all potential ligand binding sites on protein surfaces. This tool allows for different colors to be assigned to the cavities for easier visual perception. The entire graphical visualization was implemented using the JSmol subprogram on the website [77,78].

On the other hand, knowing that the intrinsic protein flexibility is relevant to the interaction with the ligand and can contribute to the binding process and that the internal cavities play an important role in this structural flexibility and stability [48], the fluctuation of the cavity volume δVV212 can be estimated using the following equation:

(2) δVV212=1N∑n=1nVVn−VV2

where $V_{V\left(n\right)}$ is the cavity volume of each true cavity estimated through the MOLE 2.5 tool, $\langle V_{V}\rangle$ is the mean value of $V_{V\left(n\right)}$, and N is the total number of cavities [48].

## 3. Results and Discussion

### 3.1. Docking, Inhibitory Potency, and MD Analysis

**Figure 1:**
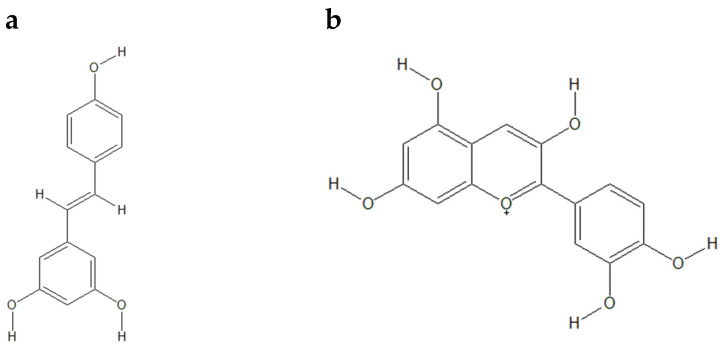

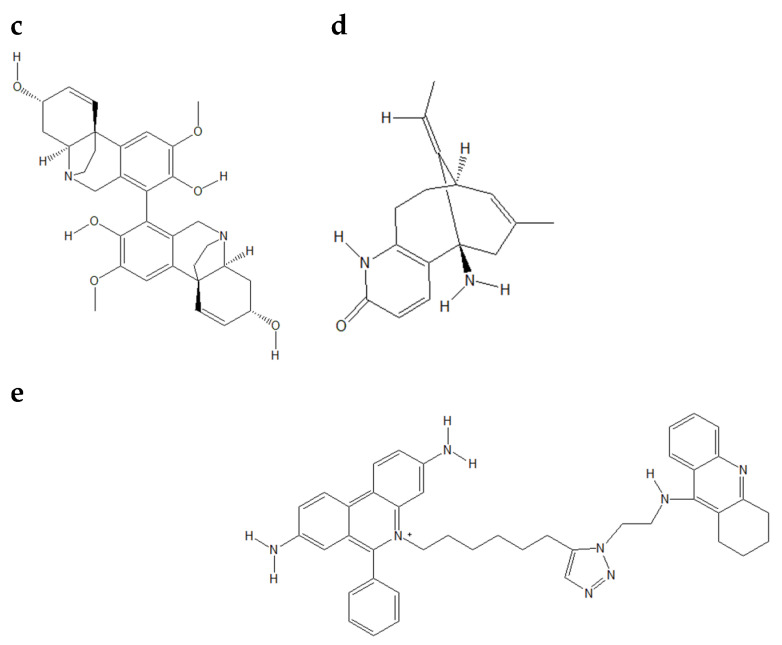
Structures of (**a**) resveratrol (CID: 445154), (**b**) cyanidin (CID: 128861), (**c**) bis-(-)-8-demethylmaritidine (CID: 169450457), (**d**) huperzine A (CID: 854026), and (**e**) TZ2PA6 (CID: 5289508) drawn using ChemSketch Freeware.

Table 1 shows the results of molecular docking obtained by machine learning using the DockThor algorithm for the compounds considered in this study against AChE (Figure 1). Additionally, it presents the values obtained from the post-classification performed to validate the thermodynamically most favorable binding interactions identified by DockThor for each complex formed between AChE and its respective inhibitor. For this validation, the MM/PBSA (molecular mechanics Poisson–Boltzmann surface area) method was employed, which was applied directly to the complex predicted by DockThor following the MD analysis conducted at 500 ns (Table 1).

**Figure 2:**
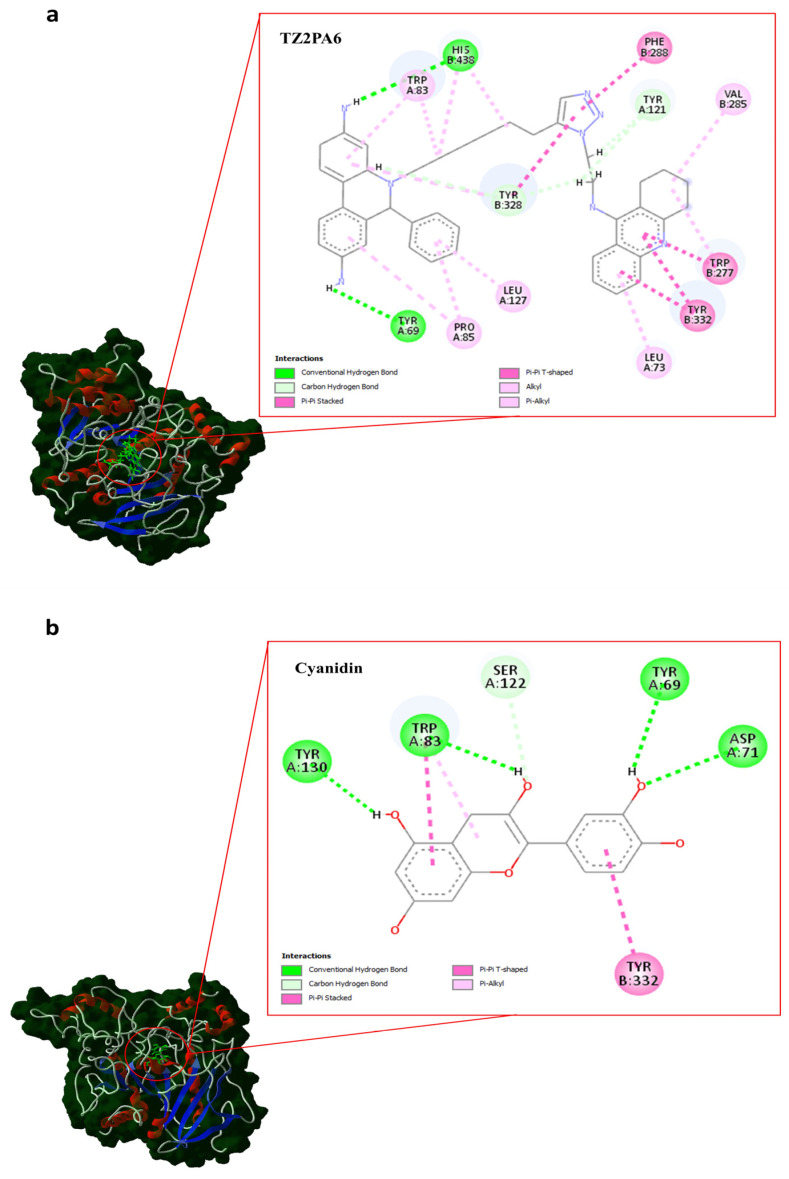
Minimum energy structures of (**a**) TZ2PA6 and (**b**) cyanidin coupled to AChE after 500 ns MD simulations.

All docking results were thermodynamically favorable, regardless of the method used for binding energy prediction (DockThor and MM/PBSA), with an overall mean binding energy of ΔG ≈ −12 kcal.mol^−1^ (Table 1). According to the DockThor calculations, the thermodynamic mean was ΔG ≈ −9 kcal.mol^−1^, with a minimum of ΔG ≈ −8 kcal.mol^−1^ and a maximum of ΔG ≈ −12 kcal.mol^−1^. TZ2PA6 was the compound with the most favorable binding free energy (ΔG = −12.03 kcal.mol^−1^), followed by cyanidin (ΔG = −9.50 kcal.mol^−1^). For illustrative purposes, the most favorable complex in each case is shown (Figure 2). The remaining compounds exhibited similar binding energies (ΔG ≈ −8.9 kcal.mol^−1^) according to the DockThor algorithm. Both TZ2PA6 and cyanidin established various interactions with AChE including hydrophobic interactions and several conventional hydrogen bonds as well as carbon hydrogen bonds. Cyanidin exhibited twice as many conventional hydrogen bonds with AChE compared to the control (TZ2PA6), which, in turn, presented double the carbon hydrogen bonds with AChE compared to those exhibited by cyanidin. It is important to note that most interactions of TZ2PA6 and cyanidin occurred with aromatic residues adjacent to the active site reported for AChE, notably highlighting the shared interaction with residues Tyr69 and Trp83 as well as with the aromatic residue Tyr332, which is also present in the active site of the highly related enzyme butyrylcholinesterase (BuChE) [79,80]. These aromatic residues are crucial for the stability of the complexes formed with different ligands [81]. These results are significant because most of the binding affinities predicted by DockThor have been described to be moderate in the range of high to low micromolar affinity units (scores < −8 kcal.mol^−1^ are associated with submicromolar affinities) [79]. In addition, the relative binding energies obtained for the compounds in this study corresponded to those reported by other authors for the same protein in the context of a wide variety of synthetic compounds and utilizing related docking tools [82,83,84].

On the other hand, the MM/PBSA calculations showed an increase in the binding energies of all compounds after MD with a global mean that increased from ΔG_MM/PBSA_ ≈ −5 kcal.mol^−1^ to ΔG_MM/PBSA_ ≈ −21 kcal.mol^−1^. The thermodynamic mean of MM/PBSA was ΔG_MM/PBSA_ ≈ −13 kcal.mol^−1^, with a minimum of ΔG_MM/PBSA_ ≈ −1 kcal.mol^−1^ and a maximum of ΔG_MM/PBSA_ ≈ −38 kcal.mol^−1^. As predicted by DockThor, TZ2PA6 was the compound with the most favorable relative binding energy calculated by MM/PBSA (ΔG_MM/PBSA_ = −38.80 kcal.mol^−1^), followed by huperzine A (ΔG_MM/PBSA_ = −22.85 kcal.mol^−1^) and cyanidin (ΔG_MM/PBSA_ = −17.25 kcal.mol^−1^) after 500 ns MD (Table 1; Figure 2). Resveratrol was one of the compounds with the least favorable relative binding energy in both the DockThor predictions and after 500 ns MD according to the MM/PBSA calculations (Table 1). These results are important because MM/PBSA calculations have been described to have an overall success rate of ≈74%, even improving the predictions made by efficient scoring functions such as Glide SP (≈59% success rate) [61].

**Figure 3:**
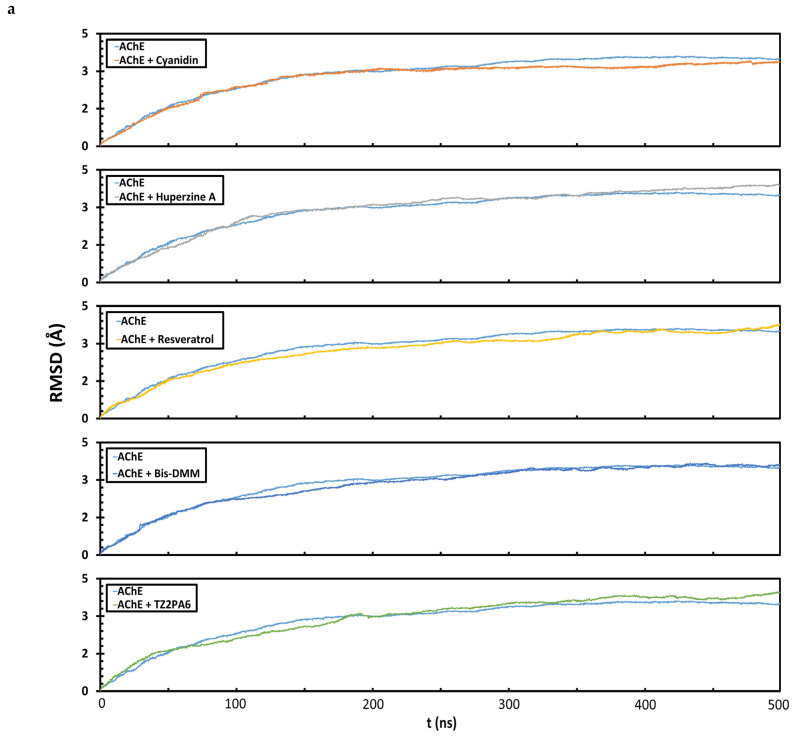

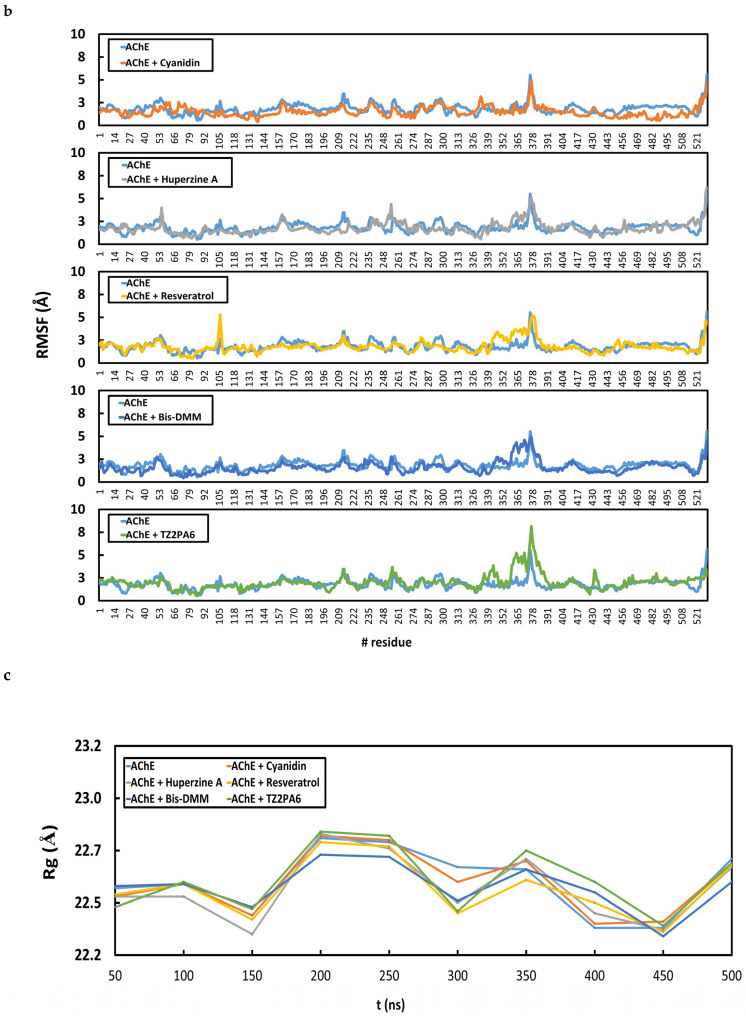

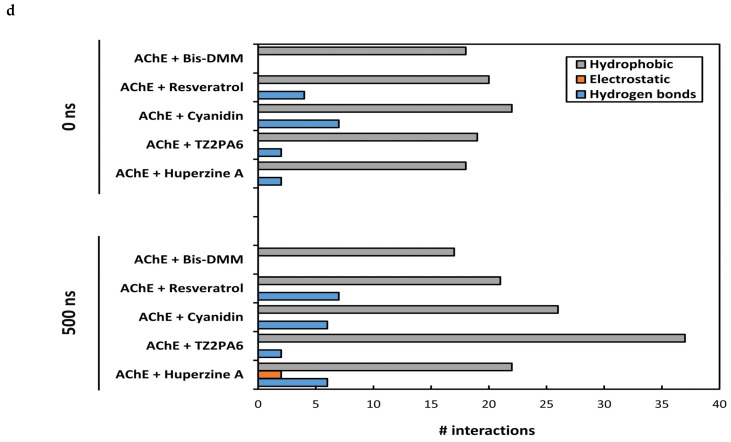
Root mean square deviation (RMSD) (**a**), root mean square fluctuation (RMSF) (**b**), radius of gyration (Rg) (**c**), and (**d**) depletion of hydrophobic, electrostatic, and hydrogen bonding interactions of minimum energy complexes AChE with and without ligands after 500 ns of MD simulations.

**Figure 4:**
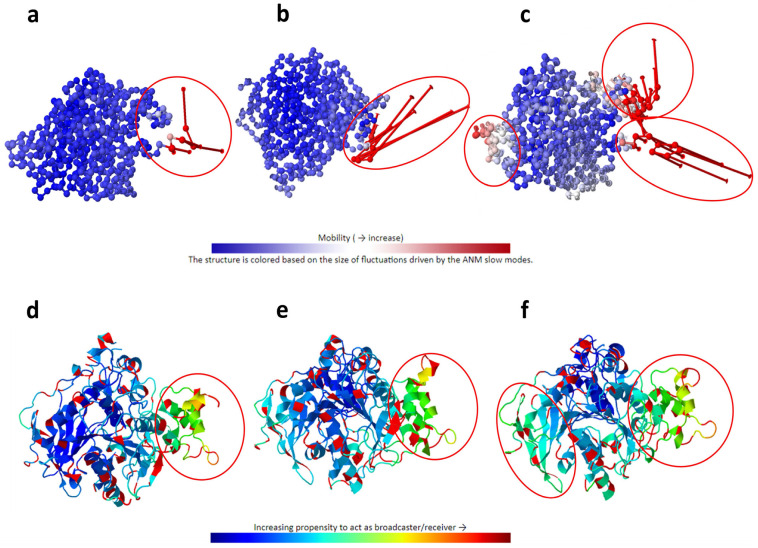
The top panel shows a graphical representation of the collective mobility of the nodes, indicated by a ribbon ranging from blue (low mobility) to red (high mobility), highlighting in a red circle parts of the complex (**a**) AChE (**b**) with TZ2PA6 and (**c**) with cyanidin after 500 ns of MD. The lower panel shows the same complexes, but this time it is represented by a ribbon ranging from blue (residues with a propensity to broadcast the response to a perturbation) to red (residues with a propensity to receive such perturbation) for the complexes (**d**) AChE, (**e**) with TZ2PA6, and (**f**) with cyanidin over the same period of time.

**Figure 5:**
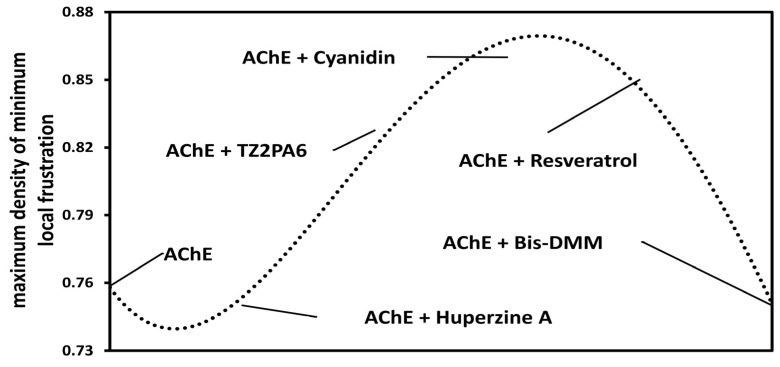
Graphical representation of the maximum values of minimally frustrated residues of the AChE protein system free and in the presence of each compound.

The complexes formed by DockThor were used in the MD simulations to evaluate the stability of the compounds bound to AChE and select low-energy conformations to study the deformability as well as the conformational and energy-structural changes of AChE in the presence of each ligand [39,68]. From the minimum energy structures generated by MD, data were obtained for (1) MM/PBSA (Table 1), (2) RMSD (Figure 3a), (3) RMSF (Figure 3b), (4) Rg (Figure 3c), and (5) interaction depletion (Figure 3d), as suggested [39,85,86]. The means of the dynamic parameters calculated after 500 ns of MD indicated the stable binding of all compounds considered in this study to AChE, regardless of the nature of the compounds (RMSD ≤ 2.8 Å, RMSF ≤ 1.7 Å, and Rg ≤ 22.55 Å) (Figure 3). Cyanidin was the compound that exhibited a slightly more stable binding oriented toward structural folding (RMSD = 2.7 Å and RMSF = 1.50 Å) compared to the free protein (RMSD = 2.9 Å and RMSF = 1.83 Å) and the other compounds (Figure 3) in terms of both the RMSD and RMSF. In all cases, an increase in the type and number of interactions was predicted after MD, with the largest number of predicted interactions being hydrophobic, except for the interactions between AChE and Bis-DMM, which showed little variability. Figure 3 displays a representative run of each trajectory at 500 ns of MD for each of the studied complexes.

We calculated the theoretical kinetic parameters of the compounds considered for which inhibition constants (Ki) or IC_50_ values against AChE have not been described (i.e., cyanidin and resveratrol) and found a higher inhibitory potency for xyanidin (pIC_50_ = 12.2) compared to resveratrol (pIC_50_ = 7.4) (Table 2). For compounds with reported experimental data for at least one of the mentioned kinetic parameters, the missing parameter was also calculated for illustrative purposes. When comparing the experimental kinetic calculations described for the rest of the compounds, it was observed that the theoretical kinetic calculations for resveratrol were like those reported experimentally for huperzine A after an average of 18 assays [46], but less favorable than those reported for TZ2PA6 [32]. The compound Bis-DMM was the compound with the least favorable experimentally reported kinetic parameters [47] (Table 2). These inhibition potency results are in line with most of the binding affinities predicted from the docking algorithm used before MD and based on machine learning, in which the compounds that presented the most favorable energies such as cyanidin and TZ2PA6 tended to also present submicromolar affinities [87].

### 3.2. Results of the Analysis of Intrinsic Deformability Associated with AChE Conformational Changes in the Presence and Absence of Compounds

In relation to the structural deformability of the complexes formed between the compounds and AChE after 500 ns of MD, data were obtained using coarse-grained computational strategies to determine possible conformational changes in these dynamic systems (Table 3) [38]. All compounds induced local changes in the residues of the AChE protein in terms of distance and solvent accessibility following the coarse-grained simulation compared to the free protein. Cyanidin was the compound that mediated the greatest perturbation of the intrinsic accessibility of the residues to the solvent compared to the rest of the complexes, and it was also able to alter the psi (Ψ) angles of the AChE system (Table 3).

On the other hand, it was additionally predicted that the greatest change induced by TZ2PA6 was in terms of the torsion of both the phi (φ) and psi (Ψ) angles, while huperzine A and resveratrol affected the psi (Ψ) angles to a greater extent (Table 3). These results show how the compounds can perturb the stability of protein biomolecules in relation to the structural fluctuations related to their energy landscape in both the free and bound states. This approach allowed us to analyze the contribution of residues to the global folding of a conformation as well as the relationship between the free energy of that conformation and the probability of observing that conformation, as has been reported [38,41].

The results obtained from the CUPSAT and SWOTein models regarding statistical potentials are novel; however, cross-validation with experimental mutagenesis data is recommended to ensure prediction accuracy and reliability concerning protein stability changes due to point mutations. The models use environment-specific atomic potentials and torsional angle potentials to predict the free energy changes between wild-type and mutant proteins. Cross-validation methods including split sampling, the jackknife method, and k-fold validation are essential for evaluating the model’s accuracy and generalizability, which are vital for drug design applications [62].

The predictions of the intrinsic quasi-rigid regions or domains (Q) showed that cyanidin, in addition to perturbing both specific angles and ASA, was the only compound that increased the number of Q regions, while the rest of the compounds were able to alter these regions but promoted a decrease in them. The increase in the predicted intrinsic Q domains in AChE in the presence of cyanidin could be related to a redistribution of the intrinsic quasi-rigid regions of AChE, indicating a probable gain in rigidity of the protein system, as opposed to the probable flexibilization of the protein by the rest of the compounds. All complexes presented valid rigidity quality (QS) values being similar or higher than those calculated for the free protein (Table 3). These perturbations in the Q regions are determinants because, thanks to the specific properties of each complex and the alterations of the free energy landscapes, they can reliably reproduce the structural fluctuations induced by the compounds. The Q region prediction method is based on the notion that, in genuinely rigid regions (validated by the internal quality parameter QS), the distances between two points remain strictly unchanged during MD [38,73].

In addition to the predicted stability at the MD level, statistical potentials, and Q regions, it was observed that all of the compounds altered the number of intrinsic nodes (#Nodes) and links (#Links) associated with the alpha carbons and their interactions in AChE, with cyanidin being the compound that mediated the greatest increase in both the number of nodes and their links compared to the rest of the complexes and free AChE (Table 3). This greater number of predicted amino acid interaction networks in the AChE + cyanidin complex has been shown to be valuable for both protein folding and establishing residue contributions to complex binding. In this sense, the stability of cyanidin binding is due to it having a greater number of interactions of the residues that act as nodes (intersecting, connecting, or binding points) [37,64].

Changes in the connectivity of nodes and links within the AChE protein system have significant implications for its function and stability, as evidenced by several studies. It has been reported that the transient formation of complexes can alter the structural topology of proteins by disrupting the structural networks of these proteins, which may influence the associated stability and functionality [88]. It has been observed that in complexes involving protein interactions, the reorganization of links during complex formation affects the distribution of central residues at the complex interfaces. These central residues are correlated with critical binding energy points, suggesting that connectivity within these networks may influence binding affinity and specificity [89]. Furthermore, for example, the architecture of ribosome-like protein networks have demonstrated how the co-evolution of these networks optimizes interconnectivity between functional centers, which is crucial for long-distance communication within proteins such as the ribosome. Therefore, the alteration of these networks may facilitate the formation of new allosteric pathways by disrupting the native behavior of the protein [90], highlighting the importance of connectivity among residues, as has also been described in cellular models [91].

To further investigate and improve the comprehensiveness of the search for possible conformational changes in the complexes, the DynOmics-ENM tool was used, which integrates the two most widely used elastic network models (GNM and ANM). After applying these ENMs to the complexes considered, it was observed in the same way that all of the compounds altered the intrinsic conformational displacement of AChE at the level of the “collectivity” data of the modes, which is indicative of the proportion of conformations that move collectively together in the same mode. Cyanidin was the compound that caused the greatest collective displacement compared to the rest of the complexes and free AChE (Table 3), promoting increased flexibility at the ends of the chain (Figure 4).

While the increased flexibility at the ends of AChE may result from the intrinsic flexibility that proteins possess at their termini, rather than a direct effect of the bound compounds—mediated by the fact that some proteins exhibit reversible folding [92]—it is important to note that, although some proteins display “promiscuous” behavior in the sense that they allow interaction or binding to multiple targets, they must also engage in specific interactions to restrict binding to only designated targets (selective promiscuity). Therefore, if a compound outside the permissible range of a protein in terms of its intrinsic selective promiscuity stably binds to that protein, circumventing various steric hindrances, it could compromise its biological activity by promoting an “induced fit” [93].

This is crucial because it has been demonstrated that induced fit can efficiently remodel the interface of a flexible receptor, converting it into an interface that is susceptible to ligand binding through the co-evolution of intermolecular contacts within the protein. From this evolutionary perspective, and perhaps more importantly, it has been observed that, unlike the conformational selection of ligands, which explains the limited changes in protein flexibility that are independent of ligand effects, induced fit, influenced by ligands in the biological context of selective promiscuity, is not restricted to a single type of ligand and is independent of intrinsic flexibility [93] In this regard, understanding the ability of ligands to induce conformational changes is important because globular proteins including AChE can undergo mechanical deformations capable of causing changes in their functional state. That is to say, if proteins are subjected to significant perturbations, they can unfold or misfold, potentially leading to a loss of their biological activity [94].

In fact, globular proteins exhibit distinct mechanical properties, particularly in terms of their critical deformation, which is a measure of the maximum strain they can withstand before yielding. Specifically, studies have demonstrated that these proteins can undergo reversible changes in response to stress; however, beyond a certain threshold—estimated to be less than approximately 5% strain—they begin to soften and exhibit viscoelastic behavior, indicating a detrimental alteration of their biological activity [95]. This is significant because our results show that both the reported inhibitor TZ2PA6 used as a control and cyanidin were capable of inducing changes, for instance, at the level of tertiary interactions and in terms of spatial distances between AChE residues, with an average change of approximately 30%, and about 20% regarding changes in the number of rigid regions of the protein, as shown in Table 3. Thus, the relationship between molecular configuration and mechanical perturbations underscores that certain proteins, such as globular proteins, exhibit significant conformational changes when subjected to disturbances exceeding their deformation tolerance limits [96].

The data suggest that the increase in the collectivity of the AChE + cyanidin complex could be associated with the stable binding of cyanidin, which promotes an increase in the local rigidity of the complex by displacing the intrinsic flexibility of the system to the ends of the chain, being able to drive the conformations of the native structure toward a folded state around the ligand as predicted in the MD analyses, and as predicted by this ENM, which considers unique characteristics, relating the environment, prediction of the functional regions of the protein, and the modeling of dynamic structures using the coarse-grained approach [74].

ENM also revealed that cyanidin was the only molecule considered in this study that slightly altered the intrinsic sensitivity (or sensor-like behavior) of the AChE protein system differently from the rest of the compounds (Figure 4). Cyanidin caused the AChE + cyanidin complex to have a greater propensity to behave as a “receptor” molecule (the greater the magnitude, the more likely a receptor-type response), that is, a greater propensity for residues to receive signals due to the effect of the ligand affecting the native contact time between the residues (Figure 4). The rest of the compounds also altered the native response of AChE to perturbations, but the measurements indicated sensor-like behavior, as in the case of the model drug TZ2PA6 (Figure 4b).

This behavior can in turn alter the system’s allosteric communication, as shown by the increased number of steps (Communication) required for the residues/nodes to transfer interaction-mediated perturbation, as indicated by the stochastic Markov model of information diffusion. This model has been developed to explore communication between residues in proteins and allows for the generation of response maps that indicate the regions where residues potentially act as sensors (capable of detecting and responding to changes in the environment) and effectors (participating in the catalysis of chemical reactions, among others) [74,79]. These observations are important because while the direct interaction of compounds like TZ2PA6 and cyanidin was predicted with residues adjacent to those described for allosteric sites 3 and 4 reported in AChE [97], changes in the allosteric communication of the system could be indicative of an indirect conformational perturbation of the topology near those sites.

Theoretical methods such as the energy frustration model allow us to locate and quantify the energy frustration present in native protein structures by comparing the contribution to the additional stabilization energy attributed to a given pair of amino acids in the native protein with the energies that would be found through a perturbation (in this case, a ligand) that is capable of creating a different environment for the pair of interacting amino acids in the protein system [45,98]. Based on the energy frustration approaches, a trend similar to that observed in previous deformability analyses was predicted, where cyanidin was the only compound whose contribution to folding led to sufficient additional stabilization for normalized native pairs of residues in terms of typical energy fluctuation (according to the global scoring criterion established by the model for minimum frustration) (Figure 5). Cyanidin managed to decrease local interaction and generate an increase in the protein regions called “minimally frustrated” or stabilized [45], as observed in terms of MD and ENM.

The reduction or minimization of energetic frustration in a protein by the action of a drug may contribute to the efficacy of inhibitors, particularly considering the role of protein flexibility in drug design and its impact on the kinetics and thermodynamics of binding. Evidence suggests that the conformational flexibility of proteins modulates both the kinetics and thermodynamics of drug binding, which is crucial for the efficacy of inhibitors [26]. Atomic-level frustration analysis has demonstrated that drug specificity is correlated with the existence of a minimally frustrated binding pocket, leading to a more directed and efficient binding landscape. This implies that reducing energetic frustration at the binding site can enhance drug specificity and affinity, thereby contributing to its efficacy [99]. Furthermore, protein flexibility allows for adaptation to different ligand conformations that can influence the kinetics of association and dissociation as well as the stability of the protein–ligand complex [26]. Incorporating protein flexibility into drug design may help identify additional interactions that prolong the drug’s residence time, thereby improving its efficacy [100].

### 3.3. Results of the Volumetric Analysis of Internal Cavities of the AChE System in the Presence and Absence of the Compounds of Interest

Cavities are internal or external molecular “voids” that act as structural determinants within proteins. These cavities are part of the external and internal spaces of biomacromolecules and facilitate processes such as the transport of substrates/products to the active sites of enzymes, the exit of by-products generated during protein synthesis at proteosynthetic centers, and more. Furthermore, they can be distributed throughout the entire biomacromolecular structure to aid in the transport of ions or molecules across cellular biomembranes [101]. Cavities are predicted using Voronoi diagrams, a method that partitions the metric space of the protein of interest based on the distances between specified discrete sets of objects. In our case, the method employs atomic centers as objects to define the cavities, with the van der Waals (vdW) radio assigned according to the parm99 force field, as has been previously implemented [102]. The molecular surface of the protein is calculated as the surface accessible to a probe with a defined probe radius (default 3 Å). A vertex of the Voronoi diagram is removed if a sphere with a specified inner threshold radius (default 1.25 Å) cannot pass through any of the sides of a predicted tetrahedron. The Voronoi diagram is partitioned into several smaller cavity diagrams, which are analyzed to identify suitable start and end points for establishing the cavity vertices [103].

**Figure 6:**
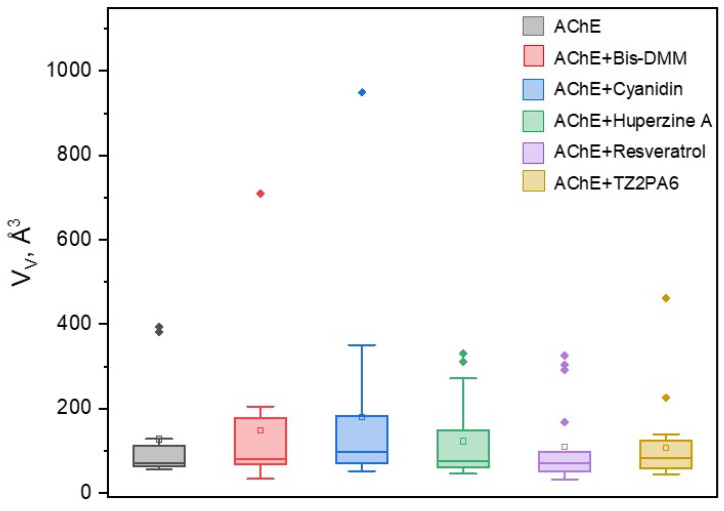
Boxplot of the distribution of the volumes of the internal cavities of the AChE protein system free and in the presence of each compound.

In this context, all compounds altered the number and distribution of AChE’s internal cavities, with an average volume of approximately 130 Å^3^. This represents a difference of approximately 5 Å^3^ compared to the average volume of AChE’s intrinsic internal cavities after 500 ns of MD, with an average minimum of approximately 30 Å^3^ and a maximum of approximately 950 Å^3^. Cyanidin was the compound that produced the greatest variability in the distribution of internal cavity volume (Figure 6), generating the largest cavities compared to the other compounds and even exceeding up to three times the volume of the largest internal cavity predicted for AChE (Table 4).

These results were further supported by the analysis of the fluctuation of the volume of the internal cavities. δVV212, which showed that cyanidin is capable of promoting an increase in almost double the fluctuation (δVV212 ≈ 222 Å^3^) compared to the native volume (δVV212 ≈ 117 Å3), followed by Bis-DMM and unlike the rest of the compounds that decreased this fluctuation (Table 4).

**Figure 7:**
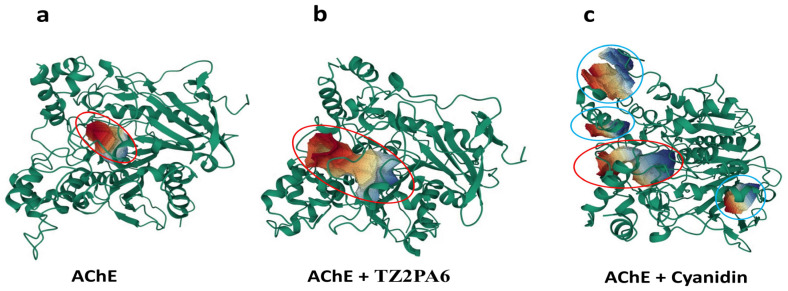
Tridimensional representation of internal cavities near the binding site. Only internal cavities with druggability probability = 1.0 are shown. In the red circle, an internal cavity near the binding pocket and the blue circles show other internal cavities in different regions with also a druggability probability = 1.0. (**a**) AChE (**b**) with TZ2PA6 and (**c**) with cyanidin after 500 ns of MD.

In this sense, when analyzing the probability that the formed internal cavities can be modulated by a drug-like molecule (druggability probability), it was found that 95% of the internal cavities generated in AChE due to the interaction with cyanidin followed by huperzine A were above the predicted probability for the rest of the compounds considered in this study. In fact, it is important to note that the internal cavities with druggability probability by the effect of cyanidin were not only those with the highest average number of residues in the site, but 75% corresponded to internal cavities with a druggability probability = 1.0, followed by 40% for TZ2PA6 (Figure 7; Table 4).

The results of this study are novel in terms of the comprehensive examination of the internal cavities conducted, particularly because most studies have provided volumetric data primarily for the catalytic pocket of acetylcholinesterase (AChE), reporting a volume of approximately 600 Å^3^. This volume is greater than those obtained in this study for all of the examined cavities, which is expected given that these are non-catalytic internal cavities [46,104]. Moreover, the druggability predictions made are important because this parameter is a key factor in determining whether a drug candidate progresses from an early stage to a more advanced stage. Therefore, druggability allows us to represent the ability of a drug to bind to cavities with high affinity [105].

Cyanidin can generate guided pockets by its proximity as well as non-guided pockets by the proximity according to the considered druggability prediction model [76]. Therefore, it could serve to mediate synergistic inhibitions as reported [106], or could be used in transport assays for other ligands by co-pigmentation using compounds even like those considered in this study [5]. These results are relevant because predicting the ability of protein cavities to bind to drugs or drug-like molecules is of great interest in the target identification phase in drug discovery or repurposing [107].

As shown by the dynamic and energy models as well as the various volumetric analysis strategies considered in this study to predict the intrinsic deformability of this type of protein system, it is evident that studying the intrinsic flexibility of AChE can be used for both system characterization and the selection of potential inhibitors of interest such as cyanidin, as has been previously conducted by applying similar approaches with the compound TZ2PA6 [30,31]. In addition, the volumetric results offer a mechanistic insight as has been suggested [48], and in turn support the observations of experimental reports demonstrating the inhibitory activity of the compounds tested in this study [32,46,47]. The results correspond with previous observations that demonstrate that inhibitory compounds can influence the volume of a protein’s internal cavity and alter its stability and conformational dynamics. The effect may vary depending on whether the inhibitor interacts with the active site or with peripheral cavities, as the latter can be crucial for functional flexibility without compromising structural stability. It has been described that inhibitors affecting peripheral cavities may have a different impact compared to those that directly target the active site, inducing complex effects that depend on the specific nature of the interaction between the inhibitor and the protein [108,109].

However, the results of this study should be taken as an approximation, as it is well-known that several conserved and structurally buried water molecules in the AChE gorge exhibit properties that may facilitate ligand entry and binding [110,111] a determining aspect that was not considered in this study. This structure, referred to as the “gorge”, has a significant impact on drug recognition due to several key features. First, this region of the active site is lined with an “aromatic patch” composed of aromatic residues such as Trp86, Tyr133, and Tyr337, which allow for versatile modes of interaction with inhibitors [112] as well as the formation of other types of stable complexes with different ligands [81]. Additionally, the conformational dynamics of the active site gorge also influence the specificity and efficiency of the enzyme. MD studies have demonstrated that the reorientation of the aromatic rings can rapidly open and close the entrance to the active site, affecting the substrate binding rate. This “breathing” capability of the gorge enables the enzyme to maintain its catalytic efficiency despite the buried location of its active site [113]. Therefore, targeting compounds to the residues of interest within the active site gorge could impact the hydrolysis of acetylcholine and offer opportunities for the selection or design of inhibitors that can interact with other sites associated with the gorge [114].

Consequently, our group is working in this direction, specifically to provide preliminary details about the likely structural and functional role that water molecules play in the modeled structures and in MD simulations, especially since it has been shown that during short simulation periods, water molecules move from the interior of the main cavity to the exterior [110]. Additionally, in order to predict stable ligand bindings based on the physicochemical perturbations of the environment, it is advisable to consider the effect of pH variations, ionic strength, and the presence of cations on protein–ligand interactions. Likewise, our group is working in this direction, considering the aforementioned parameters for computational MD simulations as well as the effect of phenomena such as cytoplasmic congestion or macromolecular crowding [38] and the comparison with other structures including those that exhibit reorganizations of the loops at the entrance of the AChE gorge [115].

Our observations demonstrate that the applied computational analyses can provide crucial insights for the design of AChE inhibitors. Techniques such as CUPSAT and SWOTein reveal how the structural attributes of AChE influence ligand binding and stability. Additionally, vibrational dynamics studies utilizing SPECTRUS and DynOmics-ENM identified conformational changes induced by various compounds, while the Frustratometer, MOLE, and PockDrug facilitated the assessment of energy frustration and the characteristics of internal cavities affected by these compounds. Collectively, these analyses indicate that cyanidin, for example, could significantly perturb AChE, highlighting its potential as an AChE inhibitor. Consequently, for experimental design and drug development, these findings suggest that understanding alterations in internal cavities can guide the rational design of novel inhibitors, paving the way for innovative therapeutic strategies targeting AChE, along with the necessary experimental validations.

Despite the novel and promising results of this study, it is important to acknowledge that these findings represent an approximation, and there are several limitations that could impact the reproducibility of the results. Firstly, the computational models employed may not fully capture the complexity of biological interactions. Additionally, the selection of a limited number of compounds hinders a broader evaluation of the diversity of potential inhibitors and their various effects. While the internal cavities of acetylcholinesterase (AChE) were analyzed, interpreting their functional relevance requires a deeper biological context. The need for the experimental validation of theoretical findings indicates that, while the inhibitory activity of cyanidin and other compounds requires confirmation, it also opens avenues for both in vitro and in vivo testing. This is particularly relevant given that, although the compounds studied here including controls with experimental evidence formed complexes with AChE through interactions with peripheral or adjacent regions to the active site, the potential to alter the biological function of this protein via quasi-allosteric pathways is useful. This consideration arises from the fact that AChE is regarded as an almost “perfect” enzyme, exhibiting a reaction rate limited by diffusion, with experimental evidence showing that only a very small fraction of substrates entering the active site gorge can react. This limitation restricts the progression rate of non-substrates of any size to the catalytic site [116]. Finally, while understanding the mechanisms of action of compounds like cyanidin is crucial, it remains a challenge to consider the pathological conditions; therefore, our group is also focused in this direction.

## 4. Conclusions

Methods such as CUPSAT and SWOTein are valuable tools in the research of inhibitors targeting AChE as they enable the evaluation of how the structural and functional characteristics of these proteins influence their interaction with ligands. These approaches facilitate the prediction of changes in AChE stability, which is crucial for the design of more effective inhibitors. Furthermore, analyses based on ENM and NMA, available in tools like SPECTRUS and DynOmics-ENM, allow for the study of the vibrational dynamics of AChE, identifying relevant conformational changes induced by the compounds, thereby optimizing the drug design strategies. Additionally, tools such as Frustratometer, MOLE, and PockDrug complement these studies by analyzing the frustration and intrinsic flexibility of AChE. The Frustratometer quantifies local frustration, aiding in the understanding of how energetic variations affect the biological function of AChE. Meanwhile, MOLE and PockDrug facilitate the identification and characterization of internal cavities, assessing their capacity to interact with drug-like molecules. Collectively, these tools provide critical insights into the dynamics and conformations of proteins, facilitating the rational design of more specific and effective inhibitors directed at AChE.

In summary, all analyses including those related to binding affinity, theoretical inhibitory potency, and dynamics demonstrated a thermodynamically favorable and stable binding of the tested compounds against AChE during the molecular dynamic simulation time considered in this study. TZ2PA6 exhibited the most favorable binding to AChE, followed by the alkaloid huperzine A and the flavonoid cyanidin. Additionally, intrinsic deformability and frustration analyses revealed that all compounds induced changes in the intrinsic flexibility and rigidity of AChE, with cyanidin causing the most significant perturbation in AChE’s structure compared to the control compounds, attributable to its in vitro inhibitory activity. Furthermore, volumetric analysis of the internal cavities indicated that while all compounds altered the distribution and size of these cavities within AChE, cyanidin generated the greatest variability in the distribution of internal cavity volume, significantly increasing the fluctuation of this volume. These findings suggest that variations in the binding affinity of the compounds may differentially impact AChE. Moreover, cyanidin may play an important role as a potential AChE inhibitor, exhibiting a distinct biophysical and molecular mechanism compared to other known inhibitors, primarily targeting alterations in the internal cavities of the AChE system. In this context, cyanidin presents a promising avenue for future research and therapeutic applications, particularly due to its ability to induce conformational changes at the level of internal cavities. This behavior is essential, as the ability of protein cavities to bind drugs is of great interest during the target identification phase of drug discovery.

## Figures and Tables

**Table 1:** Relative binding energies of the compounds bound to AChE.

Compounds	Type	kcal.mol^−1^		
		DockTScore	MM/PBSA ^a^	MM/PBSA ^b^
TZ2PA6	Click chemistry	−12.03	−9.14	−38.80
Cyanidin	Flavonoid	−9.50	−5.51	−17.25
Bis-DMM	Alkaloid	−8.97	−1.37	−16.10
Resveratrol	Polyphenol	−8.95	−3.48	−10.61
Huperzine A	Alkaloid	−8.95	−9.64	−22.85

DockTScore, scoring function calculated using the DockThor server; ^a^ 0 ns of Molecular Dynamics (MD): refers to the results at 0 nanoseconds of MD simulation; ^b^ 500 ns de MD: refers to the results at 500 nanoseconds of MD; MM/PBSA, relative binding energy calculated using the molecular mechanics with Poisson–Boltzmann (PB) method and continuous surface area solvation (SA); Bis-DMM, abbreviation for bis-(-)-8-demethylmaritidine.

**Table 2:** Theoretical inhibition of the compounds against AChE.

Compounds	µM			Ref.
	*K*_i_	IC_50_	pIC_50_	
TZ2PA6	4.0 × 10^−4^ *	8.0 × 10^−4^	9.1	[17]
Cyanidin	2.9 × 10^−7^	5.7 × 10^−7^	12.2	This work
Bis-DMM	4.0 × 10^1^	8.1 × 10^1^ *	4.1	[39]
Resveratrol	2.0 × 10^−2^	4.0 × 10^−2^	7.4	This work
Huperzine A	1.0 × 10^−2^	2.0 × 10^−2^ *	7.7	[38]

* Experimental concentration; concentration required to achieve 50% inhibition (IC_50_), and inhibitory potency expressed logarithmically (pIC_50_); Bis-DMM, abbreviation for bis-(-)-8-demethylmaritidine.

**Table 3:** Structural and conformational stability, intrinsic flexibility-rigidity (deformability) parameters using ENM, statistical potentials, and energetic frustration of AChE complexes with and without ligands after 500 ns of MD.

Complex	Distance	ASA	ϕ	Ψ	Q	QS	#Nodes	#Links	Collectivity	Receiver	*C*	*f*
AChE	−206.54	−50.23	−76.26	39.83	19	3.12	104	103	0.501	0.033	986.87	0.76
AChE + Huperzine A	−156.44	−37.90	−76.76	35.42	11	3.68	96	95	0.563	0.027	985.04	0.75
AChE + TZ2PA6	−153.51	−33.02	−77.55	37.79	4	3.29	101	100	0.564	0.031	972.16	0.83
AChE + Cyanidin	−156.22	−24.40	−75.81	37.93	21	3.38	129	128	0.584	0.035	991.64	0.86
AChE + Resveratrol	−152.60	−30.07	−75.82	34.81	17	3.02	126	125	0.532	0.028	975.85	0.85
AChE + Bis-DMM	−158.20	−27.63	−76.63	39.08	6	3.31	89	88	0.505	0.030	971.18	0.75

Bis-DMM, abbreviation for bis-(-)-8-demethylmaritidine; Distance refers to the separation between the residues of the protein and is calculated based on the average geometric centers of the heavy side-chain atoms. Values with a negative sign (−) represent stable systems in terms of statistical potentials according to SWOTein; ASA, solvent accessibility according to CUPSAT. The average values obtained for the torsion angles phi (φ) and psi (Ψ) according to the CUPSAT tool; intrinsic quasi-rigid regions or domains (Q); rigidity quality (QS) values; #Nodes, number of nodes in the system or complex; #Links, number of links that connect the nodes of the system or complex; Collectivity, where high collectivity indicates that parts of the complex move together in a specific mode; Receiver is the tendency of residues to receive signals, with lower magnitudes indicating a greater propensity for communication (*C*) or allosteric behavior according to the DynOmics-ENM model; *f*, energetic frustration.

**Table 4:** Volumetric parameters considered in this study associated with the internal cavities of AChE.

Complex		$V_{V}$ (Å^3^)			δVV212	Drugg. Prob.	Pockets Drugg Prob. = 1.0	Nb. Res.
	#Internal Cavity	Mean	Min	Max	(Å^3^)	(%)	(%)	Mean
AChE	12	127.33	56	393	117.64	94	17	19
AChE + Huperzine A	20	122.50	45	331	88.87	91	20	19
AChE + TZ2PA6	25	107.48	44	461	82.43	74	40	21
AChE + Cyanidin	15	180.07	52	949	222.91	95	75	24
AChE + Resveratrol	18	108.81	32	325	93.46	82	17	22
AChE + Bis-DMM	16	147.31	33	709	155.35	61	20	20

Bis-DMM, abbreviation for bis-(-)-8-demethylmaritidine; δVV212, fluctuation of the cavity volume estimated trough Equation (2); Drugg. Prob. = druggability probability; Pockets Drugg Prob. = pockets with a druggability probability; Nb. Res. = number of pocket residues.

## Data Availability

The original contributions presented in the study are included in the article.

## Supplementary Materials

The following supporting information can be downloaded at: https://www.mdpi.com/article/10.3390/biology13121065/s1, Table S1: Detailed parameters for the MM/PBSA calculation procedures.
