# Supplementary material for: Biological Implications of the Intrinsic Deformability of Human Acetylcholinesterase Induced by Diverse Compounds: A Computational Study
Source: Biology (Basel). 2024 Dec 19;13(12):1065. doi: 10.3390/biology13121065 (PMC11672903; doi:10.3390/biology13121065)
Supplement: Supplementary file 1 [file biology-13-01065-s001.zip › biology-3311257-supplementary.pdf]

Supplementary Material

Biological Implications of the Intrinsic Deformability of Human Acetylcholinesterase Induced by Diverse Compounds: A Computational Study

Ysaías J. Alvarado [1], Lenin González-Paz [2,*], José Luis Paz [3,*], Marcos A Loroño-González [4], Julio Santiago Contreras [4], Carla Lossada [2], Alejandro Vivas [2], Yovani Marrero-Ponce [5,6,7,8], Felix Martinez-Rios [8], Patricia Rodriguez-Lugo [1], Yanpiero Balladores [9], and Joan Vera-Villalobos [10]

The equations for calculating the MM/PBSA binding free energy are presented in equations 1 and 6:

$$\Delta G_{bind} = G_{complex} - \left(G_{receptor} + G_{ligand}\right) \quad (1)$$

$$\Delta G_{bind} = \Delta H_{bind} - T\Delta S_{bind} \quad (2)$$

$$\Delta G_{bind} \approx \Delta E_{MM} + \Delta G_{solv} - T\Delta S_{bind} \quad (3)$$

$$\Delta E_{MM} = \Delta E_{bonded} + \Delta E_{ele} + \Delta E_{vdw} \quad (4)$$

$$\Delta G_{solv} = \Delta G_{polar} + \Delta G_{nonpolar} \quad (5)$$

$$\Delta G_{nonpolar} = \gamma\left(\Delta SASA\right) + \beta \quad (6)$$

The total binding free energy (ΔG_{bind}), in this study, referred to as $\Delta G_{MM/PBSA}$, represents the difference in free energy between the bound state ($G_{complex}$) and the free state $\left(G_{receptor} + G_{ligand}\right)$. It can be further expressed as the sum of the enthalpy (ΔH_{bind}) and entropy ($-T\Delta S_{bind}$) components. In this study, we used the MM/PBSA approach to calculate the enthalpy changes, while the entropy changes were not considered due to

their computational cost and lower accuracy. The enthalpy component can be divided into the molecular mechanical energy (ΔE_{MM}) and the solvation free energy (ΔG_{solv}). The ΔE_{MM} term includes the intramolecular energy (ΔE_{bonded}), electrostatic energy (ΔE_{ele}), and van der Waals energy (ΔE_{vdW}). The ΔG_{solv} term encompasses both the polar contribution (ΔG_{polar}) and the nonpolar contribution ($\Delta G_{nonpolar}$). To calculate the energetic components and predict electrostatic solvation and free binding energies, we utilized the size-modified Poisson-Boltzmann equation (SMPBE) available at https://web.uwm.edu/smpbs/ and the APBS program accessible at https://server.poissonboltzmann.org/. Additionally, we used the parameters from the fastDRH server (http://cadd.zju.edu.cn/fastdrh/overview) [40]. The $\Delta G_{MM/PBSA}$ of the complexes was determined based on frames extracted after a 500 ns MD. For visualization and analysis, we employed MMV and BIOVIA. For more details on the method, we recommend referring to [37].

Table S1. Detailed parameters for the MM/PBSA calculation procedures

Procedure name	Radii set[1]	Polar solvation energy	Non-polar solvation energy			
			SASA[2]	$E_{nonpolar}$[3]	γ[4]	β[5]
MM/PBSA	Tan&Luo +mbondi[6]	PBSA[7]	PBSA	γSASA + β + $E_{dispersion}$[8]	0.038	-0.569

[1]Radii used for the polar solvation free energy calculation. [2]Program or method used for the solvent accessible surface area calculation. [3]Equation used for the nonpolar part of the solvation free energy calculation. [4]Surface tension term in MM/PBSA calculations (kcal.mol^{-1} Å^{-2}). [5]Offset term in MM/PBSA calculations (kcal.mol^{-1}). [6]Tan&Luo radii for the protein and mbondi radii for the ligand. [7]Calculations are conducted with the PBSA module using the “Modified Incomplete Choleski Conjugate Gradient” Poisson-Boltzmann solver. [8]$E_{dispersion}$ is calculated by a numerical determination of the solvent accessible surface area.

CUPSAT model

The energy functions are derived from mean force potentials, which are based on the inverse Boltzmann's principle, establishing a close relationship between probability densities and energies:

$$\Delta G_a = -k_B T \ln\left(\frac{g_{ij}\left(r_d\right)}{g\left(r_d\right)} \right) \tag{7}$$

In this context $g_{ij}\left(r_d\right)$ represents the radial pair distribution function between a pair of atoms *i* and *j* separated by a distance r_d. The function $g\left(r_d\right)$ describes the reference state. To construct the mean force potentials, the distribution of all 40 heavy atoms is considered, covering a radial range of 2.5 - 20 Å with a bin size of 0.5 Å. Similarly, the torsion angle potentials are derived from the distribution of angles ϕ and ψ for all amino acids. Boltzmann energy values are calculated based on this distribution, and a Gaussian apodization function is then applied to assign favorable energy values for specific combinations of ϕ and ψ angles of neighboring atoms. This effectively captures the energetically favorable orientations of these observed combinations. By utilizing these techniques, we can gain valuable insights into the distribution and energetics of different atomic interactions, enabling a better understanding of the conformational behavior and stability of the system under investigation.

$$f\left(\phi,\Psi\right) = \frac{1}{2\pi\sigma^2} A\left(\phi,\Psi\right) \tag{8}$$

Where, σ is the standard deviation and $A\left(\phi,\Psi\right)$ is the Gaussian function for the torsion angles ϕ and Ψ. The distribution of the torsion angle potential is adjusted around its peaks to accommodate torsion angle perturbations. This tapering ensures that the potential function smoothly accounts for variations in the torsion angles, allowing for a more accurate representation of the energy landscape.

$$A(\phi,\Psi) = e^{-\left(\frac{\Theta}{2\sigma^2}\right)} \tag{9}$$

Where, $\Theta = (\phi - \mu_\phi)^2 + (\Psi - \mu_\Psi)^2$.The frequencies of torsion angle counts vary, and the distribution of angle bins differs among different amino acids. To address this issue, the twist angle ranges for the 20 amino acids are individually normalized for both the ϕ and ψ angles. These normalized ranges are then utilized to calculate Boltzmann energy values for the mean force potentials of all amino acids:

$$\Delta G_{tor} = -k_B T \ln\left(\frac{g(\phi,\Psi)}{g_{ref}(\phi,\Psi)}\right) \tag{10}$$

Where, $g(\phi,\Psi)$ and $g_{ref}(\phi,\Psi)$ and are torsion angle distribution of a specific amino acid and the average distribution over all the amino acids, respectively. For more details of the method [41].

SWOTein model

SWOTein (Strengths and Weaknesses of prOTEINs) is a research tool that investigates how individual residues contribute to the overall folding free energy of proteins. It accomplishes this by utilizing statistical potentials derived from databases that incorporate structural elements, including inter-residue distances. Positive contributions to the free energy are interpreted as stability weaknesses, while negative contributions represent stability strengths. Statistical potentials have proven useful in studying various biophysical properties of proteins, such as protein-protein interactions. The SWOTein model employs knowledge-based mean force potentials derived from datasets of experimentally solved 3D protein structures, employing the principles of the inverse Boltzmann law. The central objective of this model is to establish a relationship between

the free energy of a given state and the probability of observing the system in that state. These statistical potentials are based on a coarse-grained representation of protein structures, focusing on main-chain heavy atoms and the amino acid-dependent geometric average center of side chains. In this model, the distance between two residues (*C*) serves as a conformational descriptor, while a sequence descriptor (*S*) represents characteristics such as the type of amino acid or a pair of amino acids. The folding free energy associated with the sequence-structure association (*C, S*) is defined in terms of conditional probabilities, specifically $P(C|S)$ and $P(C|S)_{ref}$, which indicate the likelihood of observing a particular conformation given the sequence in the native state and a reference state, respectively:

$$\Delta W(C,S) = -k_B T \log\left[\frac{P(C/S)}{P(C/S)_{ref}}\right] \tag{11}$$

Here, we chose the reference state as the state for which the probability to observe a given conformation is independent of the sequence:

$$P(C/S)_{ref} = P(C) \tag{12}$$

This reference state mimics the unfolded state or an average misfolded state. The folding free energy is thus finally expressed as:

$$\Delta W(C,S) = -k_B T \log\left[\frac{P(C,S)}{P(C)P(S)}\right] \tag{13}$$

The probabilities appearing in this equation are estimated from the frequencies of observation of the sequence and structure elements *S* and *C*. In terms of the number of occurrences *nS*, *nC* and *nCS* of *S* and/or *C* in the dataset and their total number *n*, the folding free energy reads as:

$$\Delta W(C,S) \simeq -k_B T \log\left[\frac{n_{CS}n}{n_C n_S}\right] \tag{14}$$

Within the SWOTein framework, various statistical potentials are utilized, including the "distance potential" which we will focus on in this discussion. The distance potential, denoted as ΔW_{dis}, specifically captures tertiary interactions and is based on the spatial distances (*d*) between protein residues. To construct the distance potential, the average geometrical centers of the heavy side chain atoms are used to compute distances, which range from 3 to 8 Å. This range is divided into 25 bins with a width of 0.2 Å. Additionally, two extra bins are included to group distances smaller than 3 Å and distances larger than 8 Å. The equation (19) governing the distance potential can be expressed as follows:

$$\Delta W_{dis}^{ij} \simeq -k_B T \log\left[\frac{n_{d_{ij}\delta_i\delta_j}n}{n_{\delta_i\delta_j}n_{d_{ij}}}\right] \tag{15}$$

Where, *i* and *j* are sequence positions separated by one residue at least. For more details of the method, we recommend [42].

Elastic Network models (ENM)

In the ENM model, the protein structure is represented using a reduced set of atoms, specifically the Cα atoms, which are treated as nodes. The interactions between pairs of nodes are described by a single-term Hooke harmonic potential. The system's overall energy is captured by a straightforward Hamiltonian:

$$E = \sum_{i\neq j} k_{ij}\left(d_{ij} - d_{ij}^{o}\right)^2 \tag{16}$$

Where, d_{ij} and d_{ij}^0 are the instantaneous and equilibrium distances between Cα-atoms i and j, respectively, whereas k_{ij} is a force constant whose definition varies depending on the type of ENM used. The Hessian matrix (H) stores the second derivatives of the harmonic potential and has dimensions of 3N × 3N. Diagonalizing this matrix yields a set of 3N-6 eigenvectors with nonzero frequencies, along with their associated eigenvalues.

ENM approaches utilize two methods for computing the cross-correlation of atomic motion. The first method, known as the "linear cutoff ENM," assumes a force constant of 1 for pairwise interactions between Cα atoms within a specified cutoff distance. Additionally, adjacent Cα atoms are assigned a higher force constant of 10. The second method, referred to as "Kovacs-ENM," adjusts the force constant based on the distance between the interacting particles:

$$k_{ij} = C\left(\frac{d_{ij}^0}{d_{ij}}\right)^6 \quad (17)$$

Where C is a constant (default value: 40 $kcal.mol^{-1}.Å^{-2}$). The cross-correlations of the trajectory movements used for filtering are obtained from the covariance matrix C (Eq. 21). The structural perturbation method with ENM is a valuable technique for characterizing allosteric wiring diagrams within the context of the lower frequency modes of ENM. This approach involves systematically perturbing all the springs that connect Cα atoms and measuring the residue-specific response to these perturbations in the context of a given m-mode. The disturbance response is calculated as:

$$\delta w_m = v_m^T.\delta H.v_m \quad (18)$$

Where, v_m is the eigenvector of mode m, v_m^T is its transpose, and δH is the Hessian matrix of the perturbation to the energy of the elastic network:

$$\delta E = \frac{1}{2}\sum_{i\neq j}\delta k_{ij}\left(d_{ij} - d_{ij}^{0}\right)^2 \quad (19)$$

The response, denoted as δw_m is directly proportional to the elastic energy of the springs connected to the ith residue when they are perturbed by a fixed value (0.1). This response helps identify the most critical nodes that contribute to the dynamics of a specific mode. In this context, a node represents a connection point or a group of vertices within the protein structure graph, typically represented by the Cα atom of a residue [45,46]. The user can specify the number of modes used for computation, ranging from 1 to 3N-6. Additionally, the involvement coefficients I between the ENM modes and the displacement vector between a given structure/frame T and a reference structure R can be calculated using the following equation:

$$I_m = \frac{\sum_{n,i=1}^{3N} v_{mn}\Delta r_i}{\sum_{n=1}^{3N} v_{mn}^2 \sum_{i=1}^{3N}\Delta r_i^2} \quad (20)$$

Where, $\Delta r_i = r_i^T - r_i^R$ and $r_i^{T,R}$ is the ith coordinate in the two conformers and v_{mn} is the nth element of eigenvector m. By default, the computation is performed for all 3N-6 modes, and only the values of I that exceed a predefined threshold (e.g., 0.2) are displayed as output. The cumulative square overlap (CSO) between all modes and the displacement vector can be calculated using the following equation:

$$CSO = \sqrt{\sum_{m=1}^{3N-6} I_m^2} \quad (21)$$

Finally, residue correlation C_{ij} is computed as:.

$$C_{ij} = \sum_{I=1}^{N}\frac{v_{iI}v_{jI}}{\lambda_I} \Bigg/ \left(\sum_{m=1}^{N}\frac{v_{im}v_{jm}}{\lambda_m}\right)^{1/2}\left(\sum_{n=1}^{N}\frac{v_{in}v_{jn}}{\lambda_n}\right)^{1/2} \quad (22)$$

Where, C_{ij} denotes the correlation between particles i and j, M is the number of modes considered for computation (the first 10 non-zero frequency modes), v_{xy} and λ_y are, respectively, the xth element and the associated eigenvalue of the yth mode [43,47-49].

Normal mode analysis (NMA)

In NMA, each normal mode consists of a strain vector, which represents the direction of atomic displacement, and a frequency, which indicates the relative amplitude of motion. This technique is commonly employed in modeling protein flexibility [50]. NMA provides a means to measure the vibrational motion of a harmonic oscillating system in close proximity to its equilibrium state. It also characterizes small amplitude movements within a potential well when the system is in equilibrium. In this context, the generalized forces acting on the system are equal to zero. At this minimum q_0, the potential energy can be expanded in a Taylor series, yielding a quadratic approximation V, to the potential energy E, with respect to the generalized coordinates q_i:

$$V = \frac{1}{2}\left(\frac{\partial^2 V}{\partial q_i \partial q_j}\right)_0 \eta_i \eta_j = \frac{1}{2} V_{ij} \eta_i \eta_j \tag{23}$$

Where, η is the deviation from the equilibrium $(q_i = q_{0i} + \eta_i)$. Similarly, the kinetic energy, τ, is also approximated as a quadratic function. The Lagrangian is given by $L = \tau - V$, which leads to the η linear differential equations of motion:

$$\tau_i \ddot{\eta}_i + V_{ij} \eta_j = 0 \tag{24}$$

By assuming an oscillatory solution, $\eta_i = a_{ik} \cos(\omega_k t + \delta_k)$ and substituting it in Eq. (32), one obtains an eigenvalue problem:

$$A^{\tau}VA = \lambda \tag{25}$$

Where, A is the matrix of the amplitudes, a_{ik} and V is the matrix of the second derivatives of the potential energy and is referred to as the Hessian. λ is a diagonal matrix, and $A^{\tau}A = I$. The pattern of motions is fully specified by the vibrational normal modes, i.e., the eigenvectors (A_k) and their associated eigenvalues (λ_k). The normal mode vectors provide information about the direction and relative displacement of each particle. However, they do not provide absolute values of displacement for individual particles. In each normal mode, all particles vibrate with the same frequency [51].

SPECTRUS model

SPECTRUS utilizes an ENM approach, which takes into account the specific properties of each protein complex and its free energy landscape. This allows for reliable reproduction of structural fluctuations [52]. Quasi-rigid domain decomposition methods are based on the concept that, for truly rigid bodies, the distances between any two constituent points remain constant during motion in space. SPECTRUS method begins by calculating the distance fluctuations between each pair of amino acids, denoted as *a* and *b*,

$$f_{a,b} = \sqrt{\left\langle d_{a,b}^2 \right\rangle - \left\langle d_{a,b} \right\rangle^2} \tag{26}$$

Where, $d_{a,b}$ is the Cα atoms distance and the angled brackets denote the average over representative conformers from available crystal structures or sampled from MD trajectories [52].

Frustratometer model

The local frustration index provided by the Frustratometer measures the contribution of a residue or pair of residues to the overall energy of each structure, particularly when they deviate from their native positions. This index effectively captures the energy variations associated with the molten globule conformations of the polypeptide chain. The local frustration index is particularly valuable for studying tertiary structures and allows for the analysis of energy variations by manipulating parameters such as the distances ($r_{i,j}$) and densities of the interacting amino acids.

$$F_{ij}^{c} = \frac{E_{i,j}^{N} - \left\langle E_{i?\,j?}^{U} \right\rangle}{\sqrt{1 / N \sum_{k=1}^{n} \left(E_{i?\,j\text{创}}^{U} - \left\langle e_{i\,,j?}^{U} \right\rangle \right)^{2}}} \tag{27}$$

When $E_{i,j}^{N} - < E_{\mathrm{i}',\mathrm{j}'}^{U} >$ the native energy would not be discriminated from the typical energy of a random interaction in the molten globule and $F_{ij}^{c} \approx 0$. This approach provides a comprehensive assessment of the native pair in comparison to a range of structural decoys that may arise during the folding process. The resulting frustration index, known as "configurational frustration," accurately quantifies the level of frustration within the system. For more detailed information about the methodology, we recommend referring to the provided references [27,29].
